# The relationship between SGLT2 inhibitors and hearing loss: a nationwide population-based retrospective cohort study

**DOI:** 10.1186/s13098-025-02026-7

**Published:** 2025-12-29

**Authors:** Chia-Huei Chu, Yi-Chao Hsu, Chang-Yin Lee, Liang-Yu Lin, Wu-Lung Chuang, Heng-Jun Lin, Cheng-Li Lin, Der-Yang Cho, Kuang-Hsi Chang

**Affiliations:** 1 Department of Otolaryngology - Head Neck Surgery, MacKay Memorial Hospital, Taipei, Taiwan; 2 Department of Audiology and Speech-Language Pathology, Mackay Medical University, New Taipei City, Taiwan; 3 Institute of Biomedical Sciences, Mackay Medical University, New Taipei City, Taiwan; 4 College of Medicine, The School of Chinese Medicine for Post Baccalaureate, I-Shou University (Yancho Campus), Kaohsiung, Taiwan; 5 Department of Chinese Medicine, E-DA Hospital, Kaohsiung, Taiwan; 6 Department of Chinese Medicine, E-DA Cancer Hospital, Kaohsiung, Taiwan; 7 Division of Endocrinology and Metabolism, Department of Medicine, Taipei Veterans General Hospital, Taipei, Taiwan; 8 Faculty of Medicine, National Yang Ming Chiao Tung University, Taipei, Taiwan; 9 Division of Endocrinology and Metabolism, Department of Internal Medicine, Changhua Christian Hospital, Changhua City, 500 Taiwan; 10 Division of Endocrinology and Metabolism, Department of Internal Medicine, Lukang Christian Hospital, Changhua County, 505 Taiwan; 11 Management Office for Health Data, Medical University Hospital, China Medical University, Taichung, 404327 Taiwan; 12 Translational Cell Therapy Center, Department of Medical Research, Medical University Hospital, Taichung, 404327 Taiwan; 13 Department of Neurosurgery, China Medical University Hospital, Taichung, 404327 Taiwan; 14 Graduate Institute of Biomedical Sciences, China Medical University, Taichung, 404327 Taiwan; 15 Department of Long-Term Care, College of Nursing, Asia University, Taichung, 413 Taiwan; 16 Center for General Education, China Medical University, Taichung, 404 Taiwan

**Keywords:** Type 2 diabetes, SGLT2 inhibitor, Sensorineural hearing loss, Population-based study, Auditory outcomes

## Abstract

**Background:**

Sensorineural hearing loss (SNHL) is a common but underrecognized complication of type 2 diabetes mellitus (T2DM). Sodium-glucose cotransporter-2 inhibitors (SGLT2i) use was associated with favorable renal and cardiovascular outcomes beyond glycemic control, but their impact on auditory outcomes remains unclear.

**Methods:**

We conducted a nationwide cohort study using Taiwan’s National Health Insurance Research Database (2000–2021). Patients with T2DM who received SGLT2i for ≥ 90 days were compared with matched non-users. Propensity score matching was applied 1:1 based on demographic, clinical, and treatment characteristics. The primary outcome was the incidence of SNHL, confirmed by audiometric testing.

**Results:**

A total of 381,592 patients with type 2 diabetes mellitus (T2DM) were included. Compared with non-users, SGLT2i users exhibited a significantly lower risk of SNHL (adjusted hazard ratios (aHRs) = 0.84; 95% CI: 0.75–0.94). Subgroup analyses confirmed a consistent lower risk across sex, age, urbanization, and comorbidity strata. Notably, long-term SGLT2i use (≥ 366 days) showed a strong dose–response association with reduced SNHL risk (aHR = 0.32; 95% CI: 0.27–0.39).

**Conclusion:**

SGLT2i use in T2DM patients is associated with a reduced risk of SNHL, particularly with sustained therapy. These findings highlight a potential association between SGLT2i use and a lower risk of auditory complications in diabetes, underscoring the importance of considering hearing health within comprehensive diabetes management.

## Introduction

Diabetes-related metabolic and vascular abnormalities can damage the cochlear microvasculature, stria vascularis, and auditory neurons, leading to progressive, bilateral, high-frequency hearing impairment [1]. Epidemiologic evidence shows that individuals with diabetes have more than twice the odds of developing hearing impairment compared with non-diabetic individuals [2, 3]. Chronic hyperglycemia, oxidative stress, inflammation, and microangiopathy are key mechanisms linking diabetes to auditory dysfunction [1, 4]. Sodium–glucose cotransporter-2 inhibitors (SGLT2i) are a newer class of antihyperglycemic agents that reduce renal glucose reabsorption in the proximal tubules, thereby improving glycemic control and promoting osmotic diuresis [5, 6]. Beyond glycemic control, large clinical trials have shown that SGLT2i use is associated with cardiovascular and renal benefits, potentially mediated by improved endothelial function, reduced oxidative stress, and attenuation of chronic low-grade inflammation [7–9]. Systemic metabolic and vascular effects observed with SGLT2 inhibitor use have been associated with a lower incidence of microvascular complications in highly metabolic organs, such as the inner ear [10–12].

Cochlea and the kidney share similar structural and functional characteristics, including dense microvascular networks and specialized ion transport mechanisms that regulate fluid and electrolyte homeostasis [10–12]. Dysregulation of these processes contributes to both diabetic nephropathy and cochlear injury. Consequently, pharmacologic agents that preserve renal microvascular function may also maintain cochlear integrity. Experimental studies have suggested that SGLT2 inhibition ameliorates oxidative damage and improves mitochondrial efficiency in neural and vascular tissues, potentially protecting against SNHL [13–15]. Despite these plausible biological links, evidence on the relationship between SGLT2i use and auditory outcomes in diabetic populations remains extremely limited.

Most previous research on diabetic complications has focused on nephropathy, retinopathy, and neuropathy [7–9, 16, 17], whereas hearing impairment has received far less attention. Furthermore, whether pharmacologic interventions that improve microvascular health can translate into lower rates of SNHL has not been evaluated in large-scale population-based studies [1, 2, 4]. Given the rising prevalence of both diabetes and age-related hearing loss worldwide [1–4, 6–9], identifying modifiable pharmacologic factors that may prevent or delay auditory decline is of considerable clinical importance. Using Taiwan’s comprehensive National Health Insurance Research Database (NHIRD), which provides longitudinal medication and diagnostic data for nearly the entire population [18], we aimed to investigate whether the use of SGLT2 inhibitors (SGLT2i) is associated with the risk of developing sensorineural hearing loss among patients with type 2 diabetes mellitus (T2DM). We hypothesized that SGLT2i, through their microvascular and anti-inflammatory effects, are associated with a reduced risk of SNHL compared with other glucose-lowering therapies [7–9, 13–15].

## Materials and methods

### Data sources

Taiwan launched the National Health Insurance (NHI) program in 1995 to provide universal healthcare coverage for all citizens, achieving a coverage rate of approximately 99% of the population by 2021. The NHI is a mandatory, single-payer system that integrates claims for outpatient visits, hospitalizations, dental services, traditional Chinese medicine, and prescription drugs. Data from this system is compiled and maintained by the National Health Research Institutes (NHRI) as the National Health Insurance Research Database (NHIRD), which contains comprehensive longitudinal information on nearly 23 million beneficiaries. The NHIRD includes detailed demographic characteristics, dates of medical visits, diagnostic codes based on the International Classification of Diseases, Ninth Revision, Clinical Modification (ICD-9-CM) and Tenth Revision (ICD-10-CM), treatment procedures, and medication records. The accuracy and validity of NHIRD data for epidemiologic research have been well established, and the database has been widely used in studies of chronic diseases and pharmacoepidemiology [18–20].

For confidentiality, all personal identifiers are encrypted before release. As the NHIRD consists of de-identified secondary data, this study was exempt from full institutional review board review in accordance with the regulations of Taiwan’s Bureau of National Health Insurance and the National Health Research Institutes.

## Study population

Patients with type 2 diabetes mellitus (T2DM) were identified from the National Health Insurance Research Database (NHIRD) using ICD-9-CM codes 250.x0 and 250.x2, as well as ICD-10-CM code E11. Eligible individuals were required to have at least one inpatient or outpatient record containing these diagnostic codes. Sodium–glucose cotransporter-2 inhibitors (SGLT2i) were first reimbursed under Taiwan’s National Health Insurance on May 1, 2016, which defined the earliest possible index date for new users. NHIRD data spanning 2000–2021 were used to provide an extensive look-back window for baseline assessment and sufficient follow-up to observe study outcomes.

Patients were excluded if their index date, defined as the initiation date of SGLT2i therapy for users and a randomly assigned, calendar-year-matched date for non-users, fell outside the range of May 1, 2016, to December 31, 2020. This restriction ensured comparable exposure opportunities between the two groups after SGLT2i reimbursement began and provided adequate follow-up before database closure in 2021, thereby minimizing potential immortal-time bias.

Additional exclusion criteria included incomplete demographic data, age < 20 years, fewer than 90 days of SGLT2i use, a pre-existing diagnosis of sensorineural hearing loss (SNHL) before the index date, with type 1 diabetes (ICD-9-CM: 250.x1, 250.x3; ICD-10-CM: E10), gestational diabetes (ICD-9-CM: 648.8; ICD-10-CM: O24.4), and secondary diabetes (ICD-9-CM: 249; ICD-10-CM: E08–E09) or a follow-up shorter than 90 days. The follow-up period was defined as the time from the index date to the earliest occurrence of [1] newly diagnosed SNHL [2], death, or [3] the study end date (December 31, 2021). Baseline comorbidities, including hypertension, stroke, chronic kidney disease, ischemic heart disease, alcoholism, asthma, chronic obstructive pulmonary disease, and ear-related disorders, were identified using ICD-9-CM and ICD-10-CM codes [19, 21–23]. Concurrent antidiabetic medications (e.g., metformin, sulfonylureas, thiazolidinediones, DPP-4 inhibitors, GLP-1 receptor agonists, and insulin) were extracted via Anatomical Therapeutic Chemical (ATC) codes and used as matching variables in the propensity-score model.

## Outcome measures

The outcome variable of this study was sensorineural hearing loss (SNHL), identified using ICD-9-CM codes: (389.10, 389.11, and 389.12) and ICD-10-CM codes (H90.3, H90.41, H90.42, and H90.5). To improve diagnostic validity, patients were required to undergo pure-tone audiometry or sound field testing (NHI claim codes: 22001 B, 22001 C, 22008 B) within 90 days to meet the inclusion criteria. The follow-up period began on the index date and ended at the first occurrence of SHNL, death, or the study’s conclusion on December 31, 2021, whichever came first.

The information on comorbidities including hypertension (ICD-9-CM: 401–405; ICD-10-CM: I10-I13, I15, N26.2), stroke (ICD-9-CM: 430–438; ICD-10-CM: G45, G46, I60-I69), intracranial injury (ICD-9-CM: 850–854; ICD-10-CM: S06), chronic kidney disease (ICD-9-CM: 585; ICD-10-CM: N18), ischemic heart disease (ICD-9-CM: 410–414; ICD-10-CM: I20-I22, I24, I25), alcoholism (ICD-9-CM: 303, 305.0; ICD-10-CM: F10.10, F10.120, F10.129, F10.20, F10.21, F10.220, F10.229), asthma (ICD-9-CM: 493; ICD-10-CM: J45), chronic obstruction pulmonary disease (COPD; ICD-9-CM: 491, 492, 496; ICD-10-CM: J41- J44), impacted cerumen (IC; ICD-9-CM: 380.4; ICD-10-CM: H61.2), suppurative and unspecified otitis media (SUOM; ICD-9-CM: 382; ICD-10-CM: H66, H67), chronic serous otitis media (CSOM; ICD-9-CM: 381.1; ICD-10-CM: H65.2), rheumatoid arthritis (ICD-9-CM: 714.0, 714.3; ICD-10-CM: M05.7-M06.0, M06.2, M06.3, M06.8, M06.9, M08) were collected for analysis. Concurrent medications, including metformin (ATC code: A10BA02, A10BD02, A10BD03, A10BD05, A10BD07, A10BD08, A10BD10, A10BD11, A10BD13-A10BD15, A10BD20), sulphonylureas (ATC code: A10BB, A10BD02), meglitinides (ATC code: A10BD14, A10BX), alpha-glucosidase inhibitors (AGI; ATC code: A10BF), thiazolidinediones (ATC code: A10BG, A10BD05, A10BD09), DPP-4 inhibitors (DPP4i; ATC code: A10BH, A10BD07-A10BD11, A10BD13, A10BD19, A10BD21, A10BD24), GLP-1 receptor agonists (GLP-1 RA; ATC code: A10BJ, A10AE54), and insulin (ATC code: A10A)] were collected for analysis as well. These comorbidities and medications were selected because they have been associated with SNHL in earlier studies [19, 21–23].

Various independent variables were assessed for their potential associations with SGLT2i use and SHL. These included demographic factors such as age (categorized as 20–54, 55–64, and ≥ 65 years), sex, urbanization level (low, medium, and high), and insurance premiums (< USD 625, USD 625–USD 1250, and > USD 1250). SGLT2i users were matched 1:1 with non-users using propensity score matching through the nearest neighbor method. Initially, matching was performed to the eighth digit, and if no match was found, it was progressively relaxed to the first digit. The matching process began with a caliper width of 0.0000001, which gradually increased to 0.1 for unmatched cases. To refine the matching, we reassessed the criteria. We conducted a rematch using a greedy algorithm, ensuring that each SGLT2i patient was paired with the closest match based on their propensity score. Standardized mean differences (SMDs) were used to evaluate covariate balance after matching, with an SMD < 0.1 indicating a negligible difference between groups.

### Statistical analysis

Continuous data were expressed as means with standard deviations, while categorical data were reported as counts and percentages. Differences in demographic characteristics between the exposed and unexposed cohorts were evaluated using standardized mean differences (SMD) [24]. An SMD of less than 0.1 was interpreted as indicating a negligible imbalance between groups. The incidence of SNHL (IR, per 1,000 person-years) was calculated for both groups. The Cox proportional hazards models were used to estimate hazard ratios (HRs) with corresponding 95% CIs.

Additionally, we conducted two further sensitivity analyses. First, a sensitivity analysis was performed excluding cases of unilateral hearing loss to ensure that the observed association between SGLT2i use and sensorineural hearing loss (SNHL) was not influenced by asymmetrical auditory outcomes. Second, a time-dependent exposure model was applied in which SGLT2i use was treated as a time-varying covariate to account for variations in drug exposure over the follow-up period. This approach allowed assessment of whether the temporal dynamics of SGLT2i initiation and continuation affected the risk estimates for SNHL.

All statistical tests were two-sided, and the significance level was set at *p* < 0.05. Data analyses were performed using SAS software, version 9.1 (SAS Institute, Cary, NC, USA).

## Results

### Baseline characteristics of T2DM patients with and without SGLT2i use

Table 1 summarizes the baseline demographic, clinical, and treatment characteristics of patients with T2DM according to SGLT2i use, both before and after PS matching. After 1:1 PS matching, 190,796 SGLT2i users and 190,796 non-users were included in each cohort, and all SMDs were < 0.1, indicating adequate balance between groups. The mean age was 59.06 ± 12.36 years in SGLT2i users and 58.84 ± 12.43 years in non-users (SMD = 0.018). Females accounted for 41.2% of the SGLT2i group and 40.2% of the non-user group. The mean follow-up duration was similar between groups (2.95 ± 1.34 years vs. 2.80 ± 1.33 years; SMD = 0.116).

Distributions of urbanization level and insurance premium were nearly identical after matching (all SMD < 0.01). The prevalence of significant comorbidities—including hypertension, stroke, chronic kidney disease, ischemic heart disease, asthma, COPD, and ear-related disorders—was comparable between SGLT2i users and non-users (all SMD < 0.02).

Regarding concomitant medications, the proportions of patients using metformin, sulfonylureas, thiazolidinediones, DPP-4 inhibitors, GLP-1 receptor agonists, and insulin were well balanced (all SMD < 0.01), confirming successful matching of treatment patterns. The mean duration of diabetes was also similar (7.81 ± 5.66 years in SGLT2i users vs. 7.55 ± 5.81 years in non-users; SMD = 0.044).

Before matching, substantial differences were observed between groups. SGLT2i users were generally younger (mean 58.3 ± 11.97 years vs. 61.9 ± 13.66 years), more likely to be male (60.6% vs. 50.7%), and had a higher prevalence of antihyperglycemic agent use, particularly DPP-4 inhibitors (71.8% vs. 20.7%), thiazolidinediones (38.1% vs. 8.9%), and metformin (96.4% vs. 54.2%). They also had a longer mean diabetes duration (8.95 ± 5.74 years vs. 5.39 ± 5.18 years). After matching, these differences were fully minimized.

**Table 1:** Baseline characteristics in T2DM patients with and without SGLT2i

Variables		After PS matching					Before PS matching				
		Non-SGLT2i (*N* = 190796)		SGLT2i (*N* = 190796)		SMD	Non-SGLT2i (*N* = 879075)		SGLT2i (*N* = 259037)		SMD
Sex	Female	76,696	40.20	78,515	41.15	0.019	433,008	49.26	102,023	39.39	0.200
	Male	114,100	59.80	112,281	58.85		446,067	50.74	157,014	60.61	
Age	20–54	66,307	34.75	64,193	33.64	0.023	244,935	27.86	92,125	35.56	0.166
	55–64	60,948	31.94	61,452	32.21	0.006	259,740	29.55	86,369	33.34	0.082
	65+	63,541	33.30	65,151	34.15	0.018	374,400	42.59	80,543	31.09	0.240
	mean, (SD)	58.84	12.43	59.06	12.36	0.018	61.93	13.66	58.3	11.97	0.282
Urbanization	Lowest	14,890	7.80	14,960	7.84	0.001	71,876	8.18	19,866	7.67	0.019
	Medium	73,546	38.55	73,452	38.50	0.001	339,732	38.65	99,144	38.27	0.008
	Highest	102,360	53.65	102,384	53.66	< 0.001	467,467	53.18	140,027	54.06	0.018
Insurance fee	< 625	42,075	22.05	41,899	21.96	0.002	218,775	24.89	52,879	20.41	0.107
(USD)	625–1250	98,665	51.71	99,026	51.90	0.004	448,716	51.04	134,784	52.03	0.020
	1250+	50,056	26.24	49,871	26.14	0.002	211,584	24.07	71,374	27.55	0.080
Comorbidites	HT	142,932	74.91	143,085	74.99	0.002	606,791	69.03	199,456	77.00	0.180
	Stroke	14,291	7.49	14,416	7.56	0.002	75,809	8.62	17,312	6.68	0.073
	II	14,508	7.60	14,758	7.73	0.005	78,715	8.95	19,005	7.34	0.059
	CKD	21,681	11.36	21,600	11.32	0.001	95,282	10.84	27,182	10.49	0.011
	IHD	63,339	33.20	63,735	33.40	0.004	279,553	31.80	92,521	35.72	0.083
	Alcoholism	2840	1.49	2886	1.51	0.002	14,923	1.70	3490	1.35	0.029
	Asthma	31,780	16.66	32,518	17.04	0.010	166,875	18.98	44,430	17.15	0.048
	COPD	34,492	18.08	35,431	18.57	0.013	198,104	22.54	47,117	18.19	0.108
	IC	22,142	11.61	22,678	11.89	0.009	117,232	13.34	31,155	12.03	0.039
	SUOM	14,345	7.52	14,780	7.75	0.009	71,102	8.09	20,375	7.87	0.008
	CSOM	1128	0.59	1148	0.60	0.001	5489	0.62	1613	0.62	< 0.001
	RA	398	0.21	430	0.23	0.004	3183	0.36	510	0.20	0.031
Medication	Metformin	181,380	95.06	181,464	95.11	0.002	476,618	54.22	249,705	96.40	1.121
	Sulphonylurea	139,962	73.36	140,094	73.43	0.002	281,392	32.01	207,155	79.97	1.103
	Meglitinides	11,159	5.85	11,003	5.77	0.003	22,738	2.59	16,589	6.40	0.185
	AGI	51,784	27.14	52,285	27.40	0.006	89,571	10.19	92,734	35.80	0.639
	Thiazolidinedione	53,182	27.87	53,297	27.93	0.001	77,787	8.85	98,708	38.11	0.735
	DPP4i	118,916	62.33	118,992	62.37	0.001	182,188	20.72	186,077	71.83	1.194
	GLP-1 RA	2804	1.47	2825	1.48	0.001	3466	0.39	6205	2.40	0.171
	Insulin	67,063	35.15	66,483	34.85	0.006	148,868	16.93	103,178	39.83	0.525
Duration of T2DM	< 7 years	93,800	49.16	87,225	45.72	0.069	583,104	66.33	96,588	37.29	0.607
	7 + years	96,996	50.84	103,571	54.28		295,971	33.67	162,449	62.71	
	mean, (SD)	7.55	5.81	7.81	5.66	0.044	5.39	5.18	8.95	5.74	0.650
Mean follow-up time, (SD)		2.80	1.33	2.95	1.34	0.116	2.87	1.37	3.01	1.35	0.108

SMD, standard mean difference; HT, Hypertension; II, intracranial injury; CKD, Chronic kidney disease; IHD, ischemic heart disease; COPD, chronic obstruction pulmonary disease; IC, Impacted cerumen; SUOM, suppurative and unspecified otitis media; RA, rheumatoid arthritis; CSOM, chronic serous otitis media

## Incidence rates and adjusted hazard ratios of SNHL by SGLT2i use and baseline characteristics

Table 2 presents the overall incidence rate (IR) of sensorineural hearing loss (SNHL) among patients with type 2 diabetes mellitus (T2DM), which was lower in SGLT2i users compared with non-users (1.06 vs. 1.25 per 1,000 person-years). After multivariable adjustment, SGLT2i use was significantly associated with a 16% reduction in SNHL risk (adjusted hazard ratio [aHR] = 0.84; 95% confidence interval [CI]: 0.75–0.94). Male patients exhibited a higher SNHL risk than females (aHR = 1.19; 95% CI: 1.06–1.33), and the risk increased progressively with age: compared with patients aged 20–54 years, those aged 55–64 years had an aHR = 1.40 (95% CI: 1.19–1.66), and those aged ≥ 65 years had an aHR = 2.16 (95% CI: 1.82–2.57). The level of urbanization and insurance premiums did not significantly influence SNHL risk. However, patients with comorbidities demonstrated a substantially higher risk than those without (aHR = 1.39; 95% CI: 1.12–1.73), and those with diabetes of more than 7 years also had an elevated risk (aHR = 1.20; 95% CI: 1.04–1.37). In terms of concurrent medication use, SGLT2 Inhibitors and metformin were associated with a modestly lower risk of SNHL. At the same time, most other antidiabetic agents, including sulfonylureas, thiazolidinediones, and DPP4 inhibitors, showed no significant effect. Alpha-glucosidase inhibitor (AGI) users had a slightly higher, though nonsignificant, risk (aHR = 1.11; 95% CI: 0.98–1.26).

**Table 2:** IRs and HRs of sensorineural hearing loss associated with SGLT2i and baseline characteristics in T2DM patients

Variables		SHL	PY	IR	Adj. HR	95% CI
SGLT2i	No	665	533,371	1.25	1.00	
	Yes	595	562,961	1.06	0.84	(0.75, 0.94)
Sex	Female	519	455,266	1.14	1.00	
	Male	741	641,066	1.16	1.19	(1.06, 1.33)
Age	20–54	250	383,067	0.65	1.00	
	55–64	365	359,760	1.02	1.40	(1.19, 1.66)
	65+	645	353,506	1.83	2.16	(1.82, 2.57)
Urbanization	Lowest	103	85,386	1.21	1.00	
	Medium	475	420,991	1.13	0.95	(0.76, 1.17)
	Highest	682	589,954	1.16	0.98	(0.80, 1.21)
Insurance fee	< 625	275	238,836	1.15	1.00	
(USD)	625–1250	663	568,044	1.17	1.12	(0.97, 1.29)
	1250+	322	289,452	1.11	1.12	(0.95, 1.32)
Comorbidites	No	91	148,703	0.61	1.00	
	Yes	1169	947,629	1.23	1.39	(1.12, 1.73)
Duration of T2DM	< 7 years	423	509,657	0.83	1.00	
	7 + years	837	586,675	1.43	1.20	(1.04, 1.37)
Medication	Metformin	1213	1,048,430	1.16	0.94	(0.69, 1.28)
	Sulphonylurea	1006	829,895	1.21	1.00	(0.85, 1.17)
	Meglitinides	70	58,949	1.19	0.86	(0.67, 1.10)
	AGI	452	315,446	1.43	1.11	(0.98, 1.26)
	Thiazolidinedione	415	319,315	1.30	0.93	(0.82, 1.06)
	DPP4i	846	685,907	1.23	1.04	(0.92, 1.18)
	GLP-1 RA	20	15,674	1.28	1.28	(0.82, 2.00)
	Insulin	504	384,019	1.31	1.03	(0.91, 1.16)

SHL, number of sensorineural hearing loss patients; PY, person-year; IR, incidence rate (per 1000 person-years); Adj. HR, adjusted hazard ratio adjusted for age, gender, urbanization, insurance fee, comorbidities, and medications; CI, confidence interval; HT, Hypertension; II, intracranial injury; CKD, Chronic kidney disease; IHD, ischemic heart disease; COPD, chronic obstruction pulmonary disease; IC, Impacted cerumen; SUOM, suppurative and unspecified otitis media; RA, rheumatoid arthritis; CSOM, chronic serous otitis media

### IRs and aHRs of SNHL associated with SGLT2i use in T2DM patients stratified by baseline characteristics (subgroup analysis)

By sex, SGLT2i users demonstrated lower adjusted HRs in both females (aHR = 0.88; 95% CI: 0.74–1.05) and males (aHR = 0.81; 95% CI: 0.70–0.93). Age-stratified analysis revealed significant protection among middle-aged patients (55–64 years; aHR = 0.72; 95% CI: 0.58–0.88), with a trend toward risk reduction in older adults (≥ 65 years; aHR = 0.88; 95% CI: 0.75–1.03). Across urbanization levels, SGLT2i use was associated with a lower risk of SNHL, particularly in medium (aHR = 0.82; 95% CI: 0.69–0.99) and highly urbanized areas (aHR = 0.86; 95% CI: 0.74–1.00.74.00). Similarly, patients in the mid-income category (insurance premium = $ 625-$ 1,250) benefited most from SGLT2i use (aHR = 0.74; 95% CI: 0.63–0.86). Patients with comorbidities retained a significantly lower risk (aHR = 0.84; 95% CI: 0.75–0.94), and this association was also evident in those with diabetes of more than 7 years’ duration (aHR = 0.82; 95% CI: 0.72–0.94). Regarding concomitant antidiabetic medications, SGLT2i users consistently exhibited lower SNHL risk across multiple drug categories, including metformin (aHR = 0.85; 95% CI: 0.76–0.95), sulfonylureas (aHR = 0.85; 95% CI: 0.75–0.96), thiazolidinediones (aHR = 0.72; 95% CI: 0.60–0.88), DPP4 inhibitors (aHR = 0.86; 95% CI: 0.75–0.98), and insulin (aHR = 0.78; 95% CI: 0.65–0.93). The most substantial effect was observed among patients taking meglitinides (aHR = 0.56; 95% CI: 0.34–0.91) Table 3. 

**Table 3:** IRs and HRs of sensorineural hearing loss associated with SGLT2i in T2DM patients stratified by baseline characteristics

Variables		Non-SGLT2i			SGLT2i users			Adj HR	95% CI
		SHL	PY	IR	SHL	PY	IR		
Sex	Female	268	217,998	1.23	251	237,268	1.06	0.88	(0.74, 1.05)
	Male	397	315,373	1.26	344	325,693	1.06	0.81	(0.70, 0.93)
Age	20–54	127	187,197	0.68	123	195,869	0.63	0.89	(0.70, 1.15)
	55–64	204	173,642	1.18	161	186,118	0.87	0.72	(0.58, 0.88)
	65+	334	172,532	1.94	311	180,974	1.72	0.88	(0.75, 1.03)
Urbanization	Lowest	56	41,622	1.35	47	43,764	1.07	0.79	(0.53, 1.16)
	Medium	254	205,721	1.24	221	215,271	1.03	0.82	(0.69, 0.99)
	Highest	355	286,028	1.24	327	303,926	1.08	0.86	(0.74, 1.00)
Insurance fee	< 625	123	115,888	1.06	152	122,948	1.24	1.10	(0.87, 1.40)
(USD)	625–1250	374	276,652	1.35	289	291,392	0.99	0.74	(0.63, 0.86)
	1250+	168	140,831	1.19	154	148,621	1.04	0.88	(0.70, 1.09)
Comorbidites	No	47	74,461	0.63	44	74,242	0.59	0.95	(0.63, 1.43)
	Yes	618	458,910	1.35	551	488,719	1.13	0.84	(0.75, 0.94)
Duration of T2DM	< 7 years	227	256,383	0.89	196	253,274	0.774	0.87	(0.72, 1.05)
	7 + years	438	276,988	1.58	399	309,687	1.288	0.82	(0.72, 0.94)
Medication	Metformin	636	508,555	1.25	577	539,875	1.07	0.85	(0.76, 0.95)
	Sulphonylurea	526	400,329	1.31	480	429,566	1.12	0.85	(0.75, 0.96)
	Meglitinides	44	28,748	1.53	26	30,201	0.86	0.56	(0.34, 0.91)
	AGI	236	149,394	1.58	216	166,053	1.30	0.84	(0.70, 1.01)
	Thiazolidinedione	234	151,619	1.54	181	167,695	1.08	0.72	(0.60, 0.88)
	DPP4i	434	326,940	1.33	412	358,968	1.15	0.86	(0.75, 0.98)
	GLP-1 RA	11	7101	1.55	9	8573	1.05	0.72	(0.29, 1.78)
	Insulin	273	183,930	1.48	231	200,090	1.15	0.78	(0.65, 0.93)

SHL, number of sensorineural hearing loss patients; PY, person-year; IR, incidence rate (per 1000 person-years); Adj. HR, adjusted hazard ratio adjusted for age, gender, urbanization, insurance fee, comorbidities, and medications; CI, confidence interval

### IRs and HRs of sensorineural hearing loss associated with days of supply of SGLT2i in T2DM patients

Table 4 presents the IRs and aHRs for SNHL associated with different durations of SGLT2i use in T2DM patients. Patients without SGLT2i exposure had an incidence rate of 1.25 per 1,000 person-years and served as the reference group (aHR = 1.00). For short-term SGLT2i use (90–180 days), the IR increased to 2.85, with an elevated risk compared to non-users (aHR = 2.35, 95% CI: 2.02–2.74). However, for intermediate use (181–365 days), the IR decreased to 1.29, indicating no significant difference from non-users (aHR = 1.08, 95% CI: 0.93–1.25). Notably, long-term use (≥ 366 days) substantially lowered the IR to 0.42 and significantly reduced the SNHL risk (aHR = 0.32, 95% CI: 0.27–0.39). These findings suggest a dose-response relationship, with prolonged SGLT2i therapy considerably decreasing the risk of SNHL in T2DM patients.

**Table 4:** IRs and HRs of sensorineural hearing loss associated with days of supply of SGLT2i in T2DM patients

Variables	SHL	PY	IR	Adj. HR	95% CI	Adj. HR	95% CI
SGLT2i drug days							
No (*N* = 190796)	665	533,371	1.25	1.00			
90–180 (*N* = 37743)	227	79,622	2.85	2.35	(2.02, 2.74)	1.00	
181–365 (*N* = 77661)	243	187,739	1.29	1.08	(0.93, 1.25)	0.45	(0.38, 0.54)
366+ (*N* = 75392)	125	295,600	0.42	0.32	(0.27, 0.39)	0.12	(0.10, 0.15)

SHL, number of sensorineural hearing loss patients; PY, person-year; IR, incidence rate (per 1000 person-years); Adj. HR, adjusted hazard ratio adjusted for age, gender, urbanization, insurance fee, comorbidities, and medications; CI, confidence interval

### Sensitivity analysis (excluding unilateral hearing loss) and Time-dependent exposure modeling

In the sensitivity analysis excluding cases of unilateral hearing loss, the protective association between SGLT2 inhibitor (SGLT2i) use and sensorineural hearing loss (SNHL) remained robust. Among non-users, the incidence rate (IR) was 1.15 per 100,000 person-years (614 events over 533,078 PY), whereas SGLT2i users exhibited a lower IR of 0.98 per 1,000 person-years (553 events over 562,641 PY). After adjustment for potential confounders, SGLT2i use was still associated with a 15% reduction in SNHL risk (adjusted hazard ratio [aHR] = 0.85; 95% confidence interval [CI]: 0.76–0.95). These findings confirm the consistency of the main results and indicate that excluding unilateral cases did not materially alter the association between SGLT2i use and reduced risk of SNHL (Table 5).

**Table 5:** Sensitivity analysis excluding unilateral hearing loss

Variables	SHL	PY	IR	Adj. HR	95% CI
SGLT2i					
No	614	533,078	1.15	1.00	
Yes	553#	562,641	0.98	0.85	(0.76, 0.95)

PY: person-years; IR: incidence rate; HR: hazard ratio; CI, confidence interval; #, (excluding unilateral hearing loss)

To further validate the robustness of the observed association, a time-dependent Cox regression model was conducted. In this analysis, SGLT2i use was treated as a time-varying covariate to account for changes in medication exposure during follow-up. Results remained consistent with the primary model: SGLT2i users demonstrated a 27% lower risk of SNHL compared with non-users (crude HR, 0.73; 95% CI, 0.65–0.82; adjusted HR, 0.73; 95% CI, 0.65–0.82). This reinforces that the association between SGLT2i use and reduced SNHL risk persisted even after considering dynamic exposure status over time (Table 6).

**Table 6:** Time-dependent exposure modeling

Variables	Crude HR	(95% CI)	Adj. HR	95% CI
SGLT2i				
No	1.00		1.00	
Yes	0.73	(0.65, 0.82)	0.73	(0.65, 0.82)

PY: person-years; IR: incidence rate; HR: hazard ratio; CI, confidence interval

## Discussion

Currently, there is limited literature linking SGLT2i use to SNHL. Most studies have focused on the favorable outcomes of SGLT2i in glycemic control, as well as their role in reducing cardiovascular and kidney-related risks [16, 17]. However, evidence specifically connecting SGLT2i to auditory function or HL remains scarce. Our study is the first to report that SGLT2i use is associated with additional favorable outcomes beyond glycemic control, potentially reducing the risk of SNHL.

This consistent association in the time-dependent model, which accounts for changes in SGLT2i exposure during follow-up, reinforces the robustness of our primary analysis and suggests that the observed relationship between SGLT2i use and lower SNHL risk was not driven by exposure misclassification or temporal bias.

The observed protective association between higher urbanization levels and lower SNHL risk may reflect differences in healthcare accessibility, socioeconomic status, and early detection rather than intrinsic environmental protection. Taiwan’s highly urbanized regions have denser distributions of hospitals and audiology clinics, facilitating earlier diagnosis and management of hearing problems. Moreover, residents in urban areas typically possess higher educational and income levels, factors linked to greater health literacy, adherence to medical therapy, and healthier lifestyles, all of which may indirectly reduce the risk of SNHL. Conversely, individuals in less urbanized or rural areas may experience delayed access to otologic or metabolic care, increased exposure to occupational and environmental noise, and less consistent diabetes control, potentially contributing to a higher incidence of hearing loss [1, 2, 4, 18]. However, the differences observed were modest, suggesting that urbanization likely acts as a proxy indicator of healthcare access and socioeconomic advantage rather than a direct biological modifier of SGLT2i effects.

The noticeable dose-dependent effect, in which more extended SGLT2i use was associated with a greater reduction in SNHL risk, supports the hypothesis that SGLT2i may have a protective role against SNHL in T2DM patients. These findings provide valuable insights for public health recommendations, potentially emphasizing SGLT2i use as part of T2DM management, especially in at-risk patients.

From a histological perspective, the renal tubules and inner ear share certain physiological similarities, such as the presence of ion transport mechanisms, which are crucial for maintaining homeostasis. Several ion channels and transporters are expressed in both the inner ear and kidneys [12]. Several ion transporters, such as Na, K-ATPase, Na-K-2Cl-Cotransporter (NKCC), H(+) -ATPase, Cl/HCO3 exchangers (pendrin), and various Cl- or K + channels, are located in the marginal cells of the stria vascularis. These transporters help maintain the ionic composition of the endolymph, which is crucial for stabilizing membrane potential and supporting auditory function [25]. Most of these channels and transporters are also found in renal tubules and are responsible for ion exchange or signaling. Theoretically, alterations in these channels or transporters could lead to simultaneous phenotypic changes in these two seemingly unrelated tissues. Previous studies have reported that SGLT2i use leads to an initial decline in renal function. However, with continued use for approximately one year, a lower risk of adverse effects on renal function begins to emerge [16, 19]. SGLT2i use has been associated with slower kidney disease progression and lower rates of clinically relevant renal events [16]. The dose-response effect observed in Table 4, in which greater reductions in SNHL risk were seen with longer SGLT2i use, supports the hypothesis that SGLT2i use was associated with favorable hearing outcomes in T2DM patients. Surprisingly, the effects of this medication on the kidneys and hearing appear to follow a similar pattern. Initial or short-term use might accelerate target organ function deterioration; however, after one year of continuous use, its protective functions gradually become apparent.

The first mammalian Na+/glucose cotransporter (SGLT) was cloned from rabbit intestine in 1987 [26]. Currently, six SGLT isoforms have been identified: SGLT1, SGLT2, SGLT3, SGLT4, SGLT5, and SGLT6 [27]. SGLT2, which is expressed in proximal renal tubules, reabsorbs approximately 90% of the glomerular filtered glucose. SGLT2i promotes natriuresis (sodium excretion) and osmotic diuresis, leading to increased excretion of glucose and water in the urine. This process not only lowers blood glucose but also alters water and electrolyte balance, potentially affecting the precise ion gradients and fluid homeostasis in the inner ear.

In this study, the observed association between SGLT2i use and a reduced risk of SNHL is noteworthy in diabetes care, as SNHL is a common and often overlooked complication of T2DM. Two types of glucose transporters have been described in the mammalian inner ear: the facilitated glucose transporter (GLUT) and the sodium/glucose cotransporter (SGLT) [28, 29]. Animal studies have shown that marginal cells in the stria vascularis of gerbils take up glucose via GLUT rather than SGLT [30]. In other words, the phenomenon that SGLT2i may initially affect hearing but offer lower risk over the long term is unlikely to be directly related to the drug or ion channel SGLT itself.

In the present study, short-term SGLT2i exposure (90–180 days) was paradoxically associated with a higher incidence of SNHL compared with non-use. In contrast, prolonged therapy (≥ 366 days) was linked to a markedly lower risk (Table 4). This biphasic pattern likely reflects a combination of transient physiological adaptation and methodological factors rather than an actual detrimental effect. Physiologically, the early phase of SGLT2i treatment induces osmotic diuresis and modest volume contraction, which may transiently reduce cochlear perfusion and disturb endolymphatic fluid balance, resulting in reversible auditory dysfunction [31, 32]. Such hemodynamic adaptation resembles the temporary decline in estimated glomerular filtration rate observed at SGLT2i initiation, which subsequently transitions to sustained renal protection [16, 19]. Given that the cochlea is highly dependent on stable microvascular circulation, similar short-term perfusion changes could momentarily impair auditory function. Alternatively, the increased short-term risk may arise from selection and measurement biases. Patients newly prescribed SGLT2i often present with more advanced or poorly controlled diabetes or concurrent comorbidities, factors independently associated with higher SNHL risk [1, 2, 4]. Early discontinuation due to adverse effects or suboptimal adherence could also concentrate vulnerable individuals within the short-term exposure group. In contrast, long-term users represent a healthier, adherent subset, the so-called “healthy-adherer” or depletion-of-susceptibles effect [33]. Moreover, residual confounding from unmeasured variables, such as glycemic variability, blood pressure fluctuations, or ototoxic drug exposure, cannot be excluded. Taken together, the transient elevation in SNHL risk during initial SGLT2i therapy likely reflects short-term physiological changes and selection bias rather than permanent auditory damage. The robust protective association observed with extended SGLT2i use (aHR = 0.34) supports the interpretation that sustained therapy showed associations with improved long-term vascular and metabolic profiles, potentially outweighing early treatment-related variability. Future investigations employing time-dependent exposure models and detailed metabolic data are warranted to elucidate these temporal dynamics and clarify causal mechanisms.

Although a direct causal relationship between SGLT2i use and SNHL reduction has not been firmly established, several plausible biological pathways are supported by prior experimental and clinical observations. First, hemodynamic regulation may play a role. SGLT2i therapy lowers intraglomerular pressure and improves systemic endothelial function, effects that extend to microvascular networks [16, 19, 34]. Because the cochlea relies on a dense microvasculature for oxygen and nutrient delivery, enhanced endothelial health and microcirculatory stability could mitigate ischemic injury and progressive hair-cell loss. Second, oxidative-stress modulation is another supported mechanism. SGLT2i have been shown to reduce reactive oxygen species and systemic inflammation [13–15], processes that contribute to diabetic microangiopathy and age-related cochlear degeneration [35, 36]. By improving mitochondrial function and redox balance, SGLT2 inhibition may attenuate oxidative damage within the stria vascularis and spiral ganglion neurons, thereby preserving auditory function. Third, neuroprotective effects of SGLT2i on the central nervous system have been documented in preclinical models, including increased expression of brain-derived neurotrophic factor (BDNF) and reduced amyloid accumulation [37–39]. Because BDNF is involved in both central and peripheral auditory neuron survival, these observations suggest a potential, albeit indirect, protective influence on auditory pathways. Taken together, these mechanisms are biologically plausible and align with the observed long-term protective association between SGLT2i use and lower SNHL risk in this study. Nevertheless, they remain hypothesis-generating; direct mechanistic evidence linking SGLT2i to auditory outcomes is currently lacking. Further experimental and longitudinal studies are needed to delineate the molecular pathways and confirm causality. In addition, although SNHL diagnoses were linked to audiometric testing codes to enhance validity, the NHIRD does not include actual audiogram data or calibration details. Therefore, a formal validation of the SNHL case definition in this database is lacking, and some misclassification bias, particularly of mild or subclinical cases, cannot be ruled out. Nonetheless, the combined use of diagnostic and testing codes likely improved specificity in identifying actual SNHL cases.

The complexity of diabetes-related complications can affect the inner ear through vascular, antioxidant, or neuroprotective pathways. Further research is needed to confirm these findings and elucidate the mechanisms underlying the observed effects. Our findings highlight important public health implications for managing SNHL risk among T2DM patients, particularly given the association between longer SGLT2i use and reduced SNHL risk. The finding that males and individuals aged > 65 years are at a higher risk of SNHL supports the need for targeted public health prevention strategies. Older T2DM patients, particularly those with comorbid conditions such as ischemic heart disease and COPD, may be particularly vulnerable to SNHL. These comorbidities affect vascular function and oxygenation, potentially worsening cochlear damage in patients with diabetes. Given that extended SGLT2i use was associated with a lower risk of SNHL, as shown in Table 4, clinicians may consider SGLT2i as a potential adjunctive therapy, requiring further confirmation.

This nationwide study had several limitations. Although it was a large-scale study, the average follow-up period was approximately three years due to the participant enrollment timeline. Therefore, further clinical and experimental studies are necessary to investigate the mechanisms linking SGLT2i to SNHL. Additionally, patients diagnosed with SNHL after the follow-up period were omitted, potentially leading to an underestimation of SNHL risk. Most participants were from areas with high or medium urbanization levels, which may have introduced surveillance bias due to differences in healthcare accessibility between urban and rural regions. However, because over 99% of the residents are enrolled in the National Health Insurance (NHI) program, which provides cost-free healthcare services across the country, including in remote areas, barriers to care are likely minimized, reducing surveillance bias [18]. Although SNHL diagnoses were based on claims data from the National Health Insurance Research Database (NHIRD) rather than direct physical examinations, they were confirmed through audiological and neurological evaluations to comply with regulations set by Taiwan’s Bureau of National Health Insurance. The NHIRD’s diagnostic and prescription data validity has been extensively verified in epidemiological studies, thereby ensuring diagnostic reliability [20]. To enhance the reliability of the study, control participants were selected using propensity score matching based on factors such as sex, age, index year, income, urbanization level, comorbidities, and medication use. However, this approach may have resulted in a control group with a comorbidity prevalence that does not fully reflect the general population, potentially underestimating the SNHL risk and obscuring its association with specific comorbidities. Furthermore, the duration of type 2 diabetes was not available in the National Health Insurance Research Database and thus could not be adjusted for in our analysis. The duration of diabetes is a well-recognized determinant of microvascular complications, including sensorineural hearing loss. The absence of this key baseline characteristic represents a potential unmeasured confounder that may influence the observed association between SGLT2i use and SNHL risk.

Despite these limitations, this study provides valuable insights into the potential protective relationship between SGLT2i use and SNHL development.

## Conclusion

While SGLT2 inhibitors have shown favorable associations with cardiovascular, renal, and now auditory outcomes, these potential benefits should be interpreted in conjunction with their class-specific risks, such as genital infections and rare events of diabetic ketoacidosis [40–43]. Therefore, SGLT2 inhibitors should not be considered a “preferred treatment option” for hearing protection based solely on observational data. Instead, they represent a therapeutic class that warrants further investigation in prospective, randomized studies to clarify both efficacy and safety across various clinical outcomes.

## Data Availability

Data were obtained from the National Health Insurance Research Database (NHIRD), managed by Taiwan’s NHI Administration. Due to the Personal Information Protection Act, the data are not publicly available but may be requested through the NHIRD website (https://dep.mohw.gov.tw/DOS/lp-2506-113.html).
